# The seasonal distribution, diel vertical distribution and feeding behavior of *Paraeuchaeta concinna* in the shallow subtropical coastal waters of eastern Hong Kong

**DOI:** 10.1186/2046-9063-8-28

**Published:** 2012-11-05

**Authors:** Chong Kim Wong, Eva Y W Yau, Alle A Y Lie

**Affiliations:** 1 School of Life Sciences, The Chinese University of Hong Kong, Shatin, New Territories, Hong Kong SAR, China

**Keywords:** *Paraeuchaeta concinna*, Seasonal distribution, Diel vertical migration, Feeding

## Abstract

**Background:**

Predatory copepods of the family Euchaetidae are widely distributed in polar, temperate, subtropical and tropical oceans. *Paraeuchaeta concinna* is the most abundant Euchaetidae in the subtropical coastal seas of Hong Kong and southern China. However, compared to Euchaetidae species in temperate and polar regions, relatively little information is available on the ecology of *P. concinna* and other Euchaetidae species in the subtropical oceans. This paper provides information on the seasonal abundance of *P. concinna* in the coastal seas of eastern Hong Kong. The diel vertical distribution of *P. concinna*, feeding behavior, and predation impact on mesozooplankton in eastern Hong Kong were also investigated.

**Results:**

*P. concinna* is most abundant in winter and spring. Their abundance decreases shoreward, and densities are generally higher in the open waters of eastern Hong Kong than in the inner parts of Mirs Bay and Tolo Harbour. *P. concinna* exhibits both diel vertical migration and diel feeding rhythms in Mirs Bay. *P. concinna* females show strong preference for the copepods of the genera *Acrocalanus*, *Paracalanus*, and *Parvocalanus*, and remove ~4% of their standing stocks daily.

**Conclusions:**

The low abundance of *P. concinna* during most of the year suggests it is not indigenous to coastal seas of eastern Hong Kong. *P. concinna* performs diel vertical migration, most likely as a strategy to avoid visual predation. Gut content analysis showed that *Acrocalanus*, *Paracalanus*, and *Parvocalanus* are highly preferred prey of *P. concinna.* A daily predation impact of ~4% of the standing stocks of *Acrocalanus*, *Paracalanus*, and *Parvocalanus* suggests that *P. concinna* may play an important role in regulating the populations of these small copepods in Mirs Bay, especially during winter and spring.

## Background

Marine calanoid copepods of the family Euchaetidae are widely distributed in polar, temperate, subtropical and tropical oceans, typically large in size (2–12 mm), and primarily predaceous [1]. Predation by Euchaetidae is known to significantly affect the abundance and distribution of mesozooplankton stocks in pelagic ecosystems [2-6]. Among Euchaetidae, the genera *Paraeuchaeta* and *Euchaeta*, which occur mainly in deep temperate and arctic waters, have been studied extensively [7-14]. Various patterns of diel vertical migration (DVM), in which animals migrate to different depths of the water column in a daily cycle, have been reported in several species. In *Paraeuchaeta norvegica*, some populations exhibit strong DVM [13,15], while others remain in deep waters throughout the day [16]. The vertical migratory behavior of carnivorous copepods is important as it can affect their diet, as well as the distribution of their prey and predators [7,17]. Compared to the large amount data from temperate and polar regions, information on the ecology of Euchaetidae species in tropical and subtropical coastal seas is scarce, despite their abundance and wide distribution [18,19].

Euchaetidae is one of the most abundant groups of large predatory copepods in the coastal waters of Hong Kong and southern China [20-23]. In Hong Kong, four species of Euchaetidae, including *Paraeuchaeta concinna* (also known as *Euchaeta concinna*)*, Euchaeta indica, Paraeuchaeta plana* (also known as *Euchaeta plana*), and *Euchaeta rimana*, have been reported, and their abundance is highest in oceanic waters in the east and southeast, where the influence of discharge from the Zhujiang River is low [20,24].

Tolo Harbour and Mirs Bay are two semi-enclosed bays in the northeastern part of Hong Kong. Tolo Harbour, a landlocked bay with an average depth of ~ 12 m, is connected to Mirs Bay by a narrow channel. Mirs Bay, a much larger bay with an average depth of ~ 18 m, is directly exposed to currents and waves from the open sea areas in the east and southeastern part of Hong Kong. Tolo Harbour has a copepod community of higher density and lower diversity compared to Mirs Bay [25]. The copepod communities in both bays are dominated by small copepods of the genera *Paracalanus* and *Oithona*, while larger copepods such as *Subeucalanus subcrassus* and *Calanus sinicus* are common only in the deeper waters outside Tolo Harbour [25,26]. Of the four species of Euchaetidae reported in Hong Kong, *P. concinna* is the most abundant [24]. This study aims to provide quantitative information on the seasonal distribution and feeding behavior of this predatory copepod in the coastal seas of eastern Hong Kong.

## Methods

### Seasonal distribution

**Figure 1:**
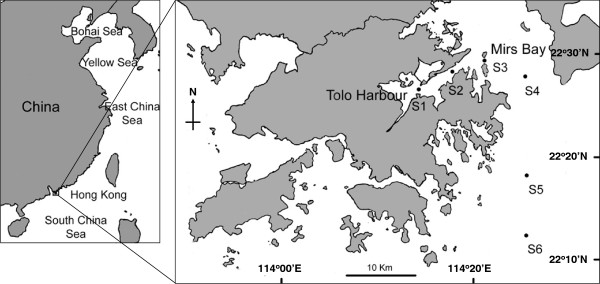
Map of Hong Kong showing the sampling stations in Tolo Harbour (S1, S2), Mirs Bay (S3, S4) and southeastern Hong Kong waters (S5, S6).

Zooplankton samples were collected at intervals of two to three weeks at six fixed stations in the eastern part of Hong Kong over a two-year period from July 2003 to June 2005 (Figure 1). S1 (22°26'725"N, 114°14'566"E) and S2 (22°28'539"N, 114°18'034"E) were located inside Tolo Harbour. S3 (22°29'468"N, 114°21'588"E) was in the inner part of Mirs Bay, near the mouth of Tolo Harbour. S4 (22°26'670"N, 114°26'920"E) was located in Mirs Bay. S5 (22°17'560"N, 114°26'920"E) and S6 (22°13'000"N, 114°26'920"E) were located outside Mirs Bay in the southeastern part of Hong Kong. Water depth increased from ~20 m at S1 to ~30 m at S6. Duplicate zooplankton samples were collected at each station by making vertical hauls from 2 m above the bottom to the surface with a conical plankton net (0.5 m mouth diameter, 125 μm mesh size) and immediately preserved in 4% buffered formaldehyde solution. All samples were collected between 1000 and 1600 h to minimize diurnal variations in zooplankton abundance. Temperature and salinity were measured at the surface (0.5 m) of each station at the time of zooplankton sampling with a Hydrolab H20 water analysis system. In the laboratory, zooplankton samples were examined and enumerated under a stereomicroscope. *P. concinna* was identified according to Park [1].

### Diel patterns in vertical distribution and gut fullness

The diel vertical distribution of *P. concinna* was studied at S5 (22°17'560"N, 114°26'920"E) on 6–7 January 2005 (Figure 1). Sunrise and sunset times were 0700 h and 1800 h, respectively. Duplicate zooplankton samples were taken from 0–5 m, 5–10 m, 10–15 m, 15–20 m and 20–25 m at 1200 h, 1600 h, 2000 h, 2400 h, 0400 h and 0800 h using a plankton net with a closing device (0.5 m mouth diameter, 125 μm mesh size). All samples were immediately preserved in 4% buffered formaldehyde solution. Vertical profiles of temperature, salinity and DO were measured at noon and midnight with a Hydrolab H20 water analysis system. Light intensity was measured at noon only with a light meter (LI-250 light meter, LI-193SA underwater quantum sensor) and was expressed as the light refractive index, which represents the ratio of light intensity in the water to light intensity just above the surface.

In the laboratory, *P. concinna* was identified according to Park [1] and densities of adults were estimated by counting entire samples. Male and female *P. concinna* were identified according to the morphological characteristic of their reproductive structures. Females with (ovigerous) and without egg sac (non-ovigerous) were counted separately. Zooplankton densities were estimated by counting at least 5% of each sample. At least 100 copepods from each sample were identified to the genus level according to the descriptions of Zheng and Zhang [19] to provide information on species compositions.

The entire digestive tracts of 10 randomly sorted *P. concinna* adult females were dissected under a stereomicroscope according to the method of Øresland [9]. The stomach content in the anterior part of each digestive tract was examined under a light microscope and prey items were identified as far as possible to the lowest taxon, using identifiable structures such as the mandibles. The gut fullness index was estimated as the ratio of the volume of stomach content to the total volume of the stomach.

### Data analysis

To compare the vertical distribution of the predator and prey, the weighted mean depth (WMD) was calculated using the equation of Worthington [27]:

$$WMD=\left(\Sigma D_{i}d_{i}\right)/\Sigma D_{i}$$

where d_*i*_ is the depth from which sample *i* was collected, and D_*i*_ is the density of *P. concinna* in sample *i*.

As Euchaetidae adult males, with their reduced mouthparts and intestines, do not feed [28,29], investigation of the feeding behavior of *P. concinna* was limited to the adult females.

Prey selectivity was calculated using the electivity measure (α) of Chesson [30]. Electivity for prey item *i* (α_*i*_) was calculated using the equation:

$$\alpha_{i}=\left(r_{i}/p_{i}\right)/\left(\Sigma r_{i}/p_{i}\right)$$

where r_*i*_ and p_*i*_ represent the proportion of prey item *i* in the gut of the predator and in the water column, respectively. The measure (α) was converted to an electivity index (ε) using the equation [31]:

$$\varepsilon_{i}=\left(m\alpha_{i}-1\right)/\left[\left(m\alpha_{i}-2\right)\alpha_{i}+1\right]$$

where m is the number of prey items and the value of ε ranges from −1 to +1. Positive values indicate selection for and negative values indicate selection against the prey item.

The feeding rate (I_*t*_) of *P. concinna* females for sampling time interval *t*, which was a duration of 4 h, was estimated by the equation:

$$I_{t}=4G_{t}/k$$

where G_*t*_ is the number of prey per female for *P. concinna* collected at time interval *t* and *k* is the digestion time in hours.

The daily predation impact (PI) of female *P. concinna* on a particular prey was then estimated using the equation:

$$PI=\Sigma \left[\left(I_{t}N_{t}/P_{t}\right)100\right]$$

where I_*t*_ is the feeding rate of female *P. concinna* at time interval *t*. N_*t*_ and P_*t*_ represent the densities of female *P. concinna* and prey at time interval *t*, respectively.

### Measurement of digestion time

Digestion time, or the time required by *P. concinna* females to digest natural copepod prey, was determined in the laboratory. Live copepods were collected by vertical hauls at S5 and immediately returned to the laboratory in surface seawater in 10-L plastic bottles. Adult females of *P. concinna* were sorted with wide-bore pipettes and maintained without food in 125 μm-filtered surface seawater at 20°C in large aquaria. After 24 h, females that still swam actively were transferred individually to 1-L fleakers. After an acclimation period of ~1 h, 30 small copepods were added to each fleaker as prey. Two groups of natural copepod prey, *Paracalanus*/*Parvocalanus* and *Acrocalanus*, were used. Feeding activities of *P. concinna* was observed directly with a magnifying lens. To facilitate the observation of prey ingestion, prey were stained with neutral red for ~30 min and washed with 125 μm filtered seawater before being fed to *P. concinna*. Females that ingested a prey were immediately transferred to fleakers without prey and remained under constant observation. Digestion time was recorded as the duration between prey capture and egestion of a colored fecal pellet by the predator. The digestion times for each group of prey were obtained by observing 10 females.

### Statistical analysis

Two criteria were used to detect the occurrence of diel vertical migration (DVM) in *P. concinna* and other copepods. Day-night differences in the WMD and percentage of population at the upper-most layer were compared using the Mann–Whitney rank sum test. Significant differences (*p* < 0.05) in both criteria indicated strong DVM, while a significant difference in only one criterion indicated weak DVM. No significant difference suggested that the animals did not exhibit DVM.

Gut fullness values were arcsine transformed and two-way ANOVA with Tukey multiple comparison was used to test for differences in the gut fullness of *P. concinna* females between day and night and among different depth layers.

## Results

### Seasonal hydrography

**Figure 2:**
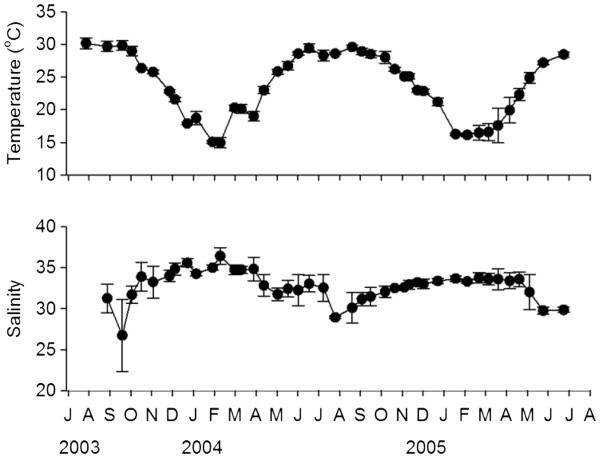
**Seasonal variations in surface water temperature and salinity.** Values are mean (± Standard deviation) of the six sampling stations.

Surface temperature was higher in summer (June to September) than in winter (December to March) (Figure 2). Surface salinity was affected by seasonal patterns in precipitation, and was lower in spring and summer (April to September) than in autumn and winter (October to March).

### Seasonal abundance and spatial distribution

**Figure 3:**
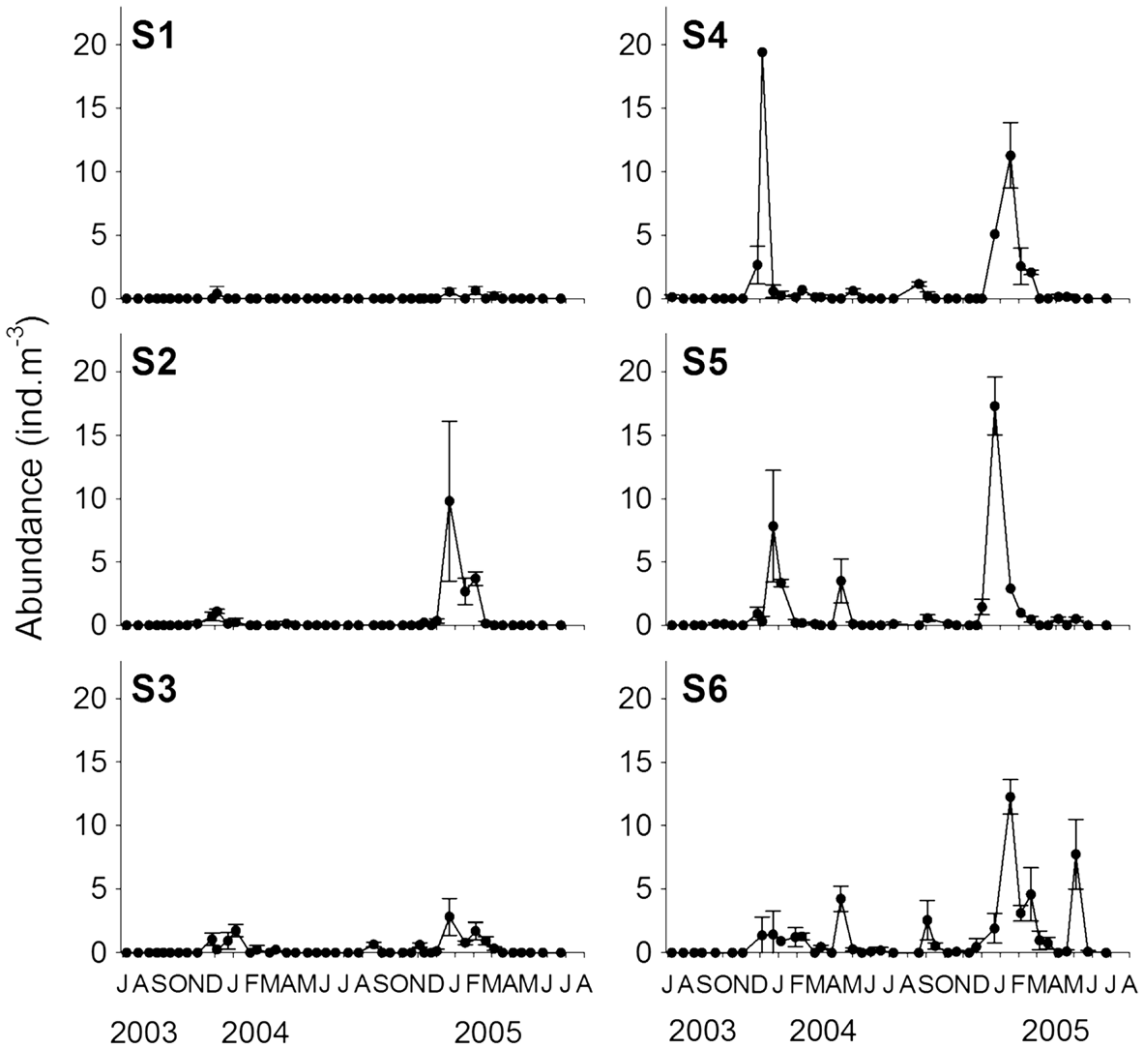
**Temporal variation in the mean density (± Standard deviation) of *P. concinna *in Tolo Harbour (S1 & S2) and Mirs Bay (S3 & S4) and southeastern Hong Kong waters (S5 & S6).**

**Figure 4:**
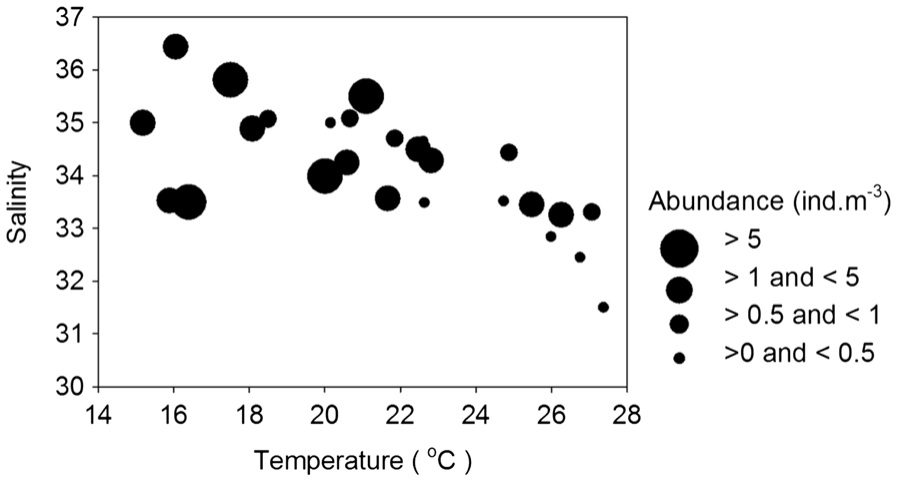
**Relationship between the density of *P. concinna *and surface water temperature and salinity.** Size of the circles represents the density range.

The density of adult *P. concinna* was lowest at S1 in the inner part of Tolo Harbour and highest at S5 and S6 in the open seas of southeastern Hong Kong (Figure 3). Over the entire study period, average densities were < 0.1–0.4 ind.m^-3^ at S1, S2 and S3, and 1.0–1.2 ind.m^-3^ at S4, S5 and S6. *P. concinna* was common only during winter and spring, and the highest densities were recorded in December and January. Density > 5 ind.m^-3^ appeared only when water temperature was < 21°C (Figure 4).

### Diel vertical distribution

**Figure 5:**
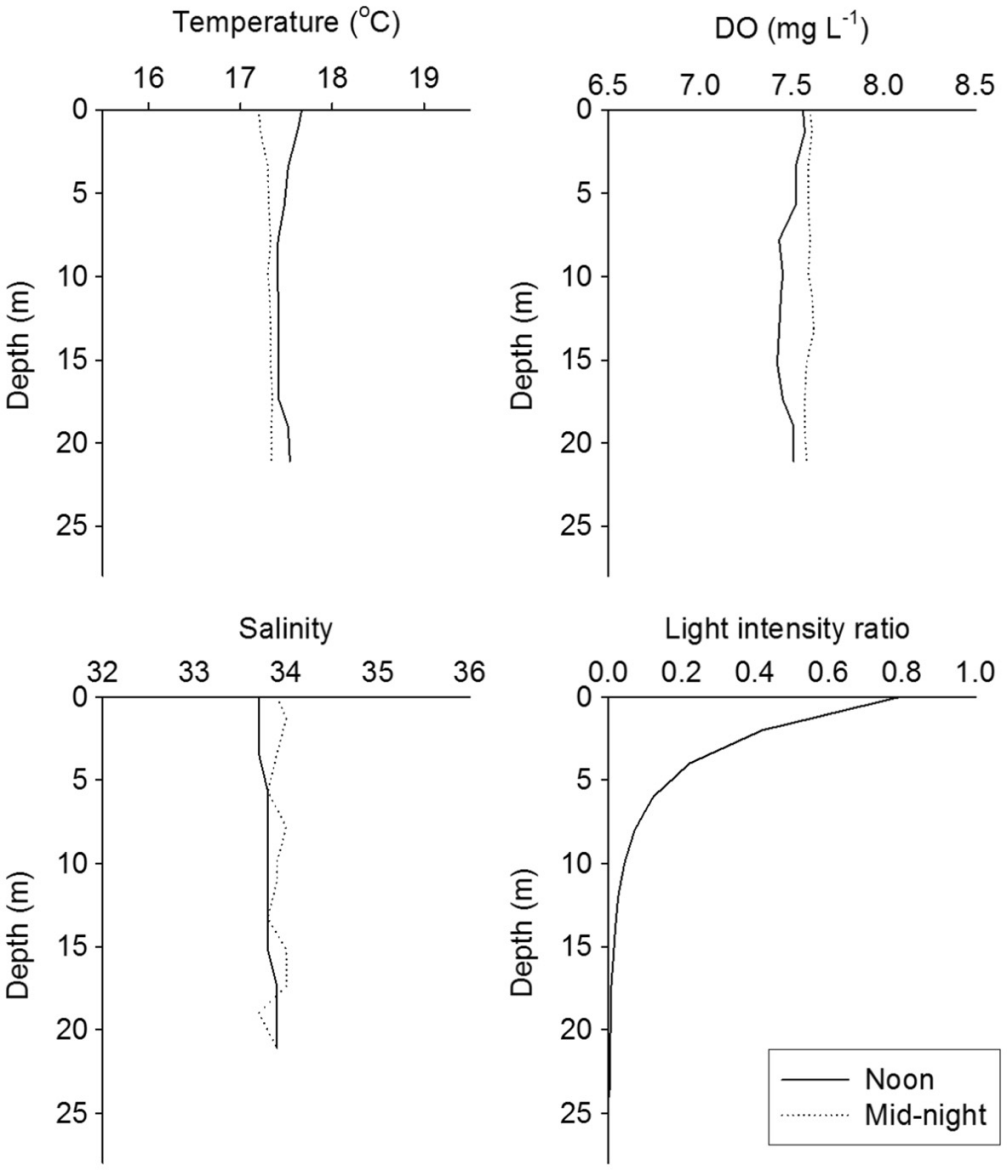
**Vertical profiles of temperature, salinity, dissolved oxygen (DO), and light intensity ratio (noon only) at noon and midnight during the diel behavior study at S5 on 6–7 January 2005.** “Light intensity ratio” refers to the ratio of light intensity in the water to light intensity just above the surface.

The diel pattern in the vertical distribution of *P. concinna* was studied at S5 on 6–7 January 2005. Averaged over the entire water column, the temperature was 17.6°C during the day and 17.3°C at night (Figure 5). The difference in temperature between surface and 20 m was slight, and the water column was well oxygenated. Light intensity was measured at noon. The ratio of light intensity in the water to light intensity above the surface revealed that light attenuated rapidly in the upper 5 m, and very little light reached depths below 15 m (Figure 5).

**Figure 6:**
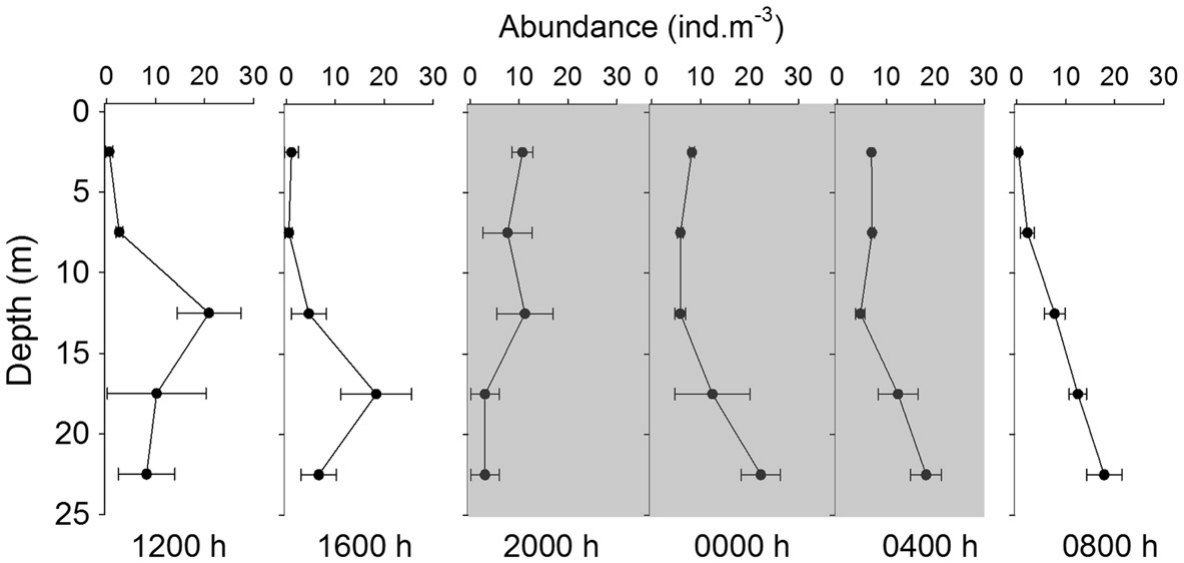
**Diel variations in the mean density (± Standard deviation) of female *P. concinna *at different layers of the water column.** Grey shading represents nighttime.

Most adult females stayed at depths below 10 m at noon and 1600 h (Figure 6). Upward migration between 1600 h and 2000 h was confirmed by an increase in density at 0–5 m from < 1 ind.m^-3^ to ~ 10 ind.m^-3^. At midnight, the density at the upper-most layer was still ~ 10 ind.m^-3^, but most of the females had returned to depths below 15 m. At 0800 h in the morning, almost no females were found at depths above 10 m. The diel vertical distribution patterns displayed by both ovigerous and non-ovigerous females were similar to that observed in the entire female population. The vertical distribution of the males was also similar to that of the females. In conclusion, all *P. concinna*, including females (both ovigerous and non-ovigerous) and males, performed DVM, as both WMD and the percentage of the population at the surface showed significant differences between day and night (*p* < 0.05) (Table 1).

**Table 1:** **Average values of weighted mean depth (WMD) and the percentage of the population at the surface during day and night for female (total, ovigerous, and non-ovigerous) and male *P. concinna*, *Acrocalanus*, *Paracalanus *and *Parvocalanus*, and *Canthocalanus *copepodites**

	**WMD (m)**			**% of population at the surface (0–5 m)**		
	**Day**	**Night**	***p***	**Day**	**Night**	***p***
Female *P. concinna*	16.8 (±1.3)	12.8 (±3.1)	<0.05	6.1 (±2.8)	38.3 (±12.1)	<0.01
Ovigerous	17.2 (±1.9)	12.9 (±3.2)	<0.05	4.1 (±5.6)	37.3 (±14.5)	<0.01
Non-ovigerous	16.9 (±1.4)	13.3 (±3.1)	<0.05	6.1 (±7.4)	33.4 (±9.6)	<0.01
Male *P. concinna*	16.0 (±1.4)	12.9 (±3.1)	<0.05	15.5 (±16.4)	40.6 (±20.7)	<0.05
*Acrocalanus*	9.8 (±3.4)	10.5 (±2.9)		55.8 (±23.6)	54.0 (±16.7)	
*Paracalanus* and *Parvocalanus*	9.9 (±1.1)	10.3 (±7.2)		56.6 (±6.1)	54.2 (±11.1)	
*Canthocalanus* copepodites	15.5 (±0.8)	12.2 (±13.4)		21.8 (±7.6)	41.2 (±11.5)	<0.05

Student’s t-test was used to test for the presence of diel vertical migration.

### Feeding and predation impact

**Figure 7:**
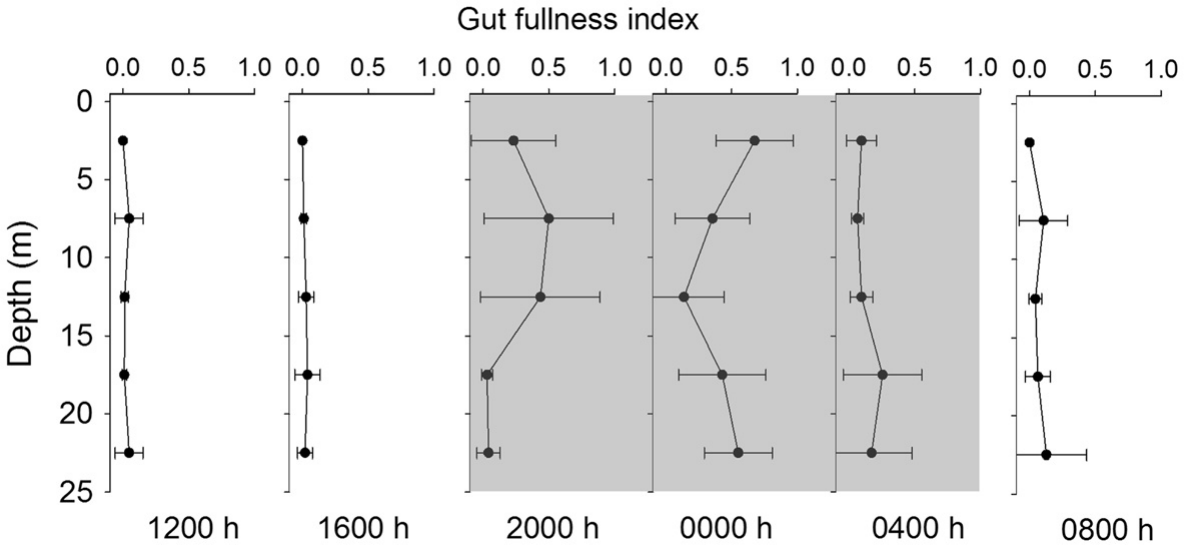
**Diel variations in the gut fullness index (± Standard deviation) of female *P. concinna *at different layers of the water column.** Grey shading represents nighttime. n = 10 for each data point.

*P. concinna* females tended to have empty guts during the day (Figure 7). Females with high gut fullness index first appeared at 2000 h, and females with the highest gut fullness index were collected at midnight at 0–5 m. At 2000 h, females collected in the upper 15 m of the water column had much higher gut fullness than females collected from the bottom. Females with high gut fullness index appeared in the bottom layers at midnight and 0400 h. These observations suggested that *P. concinna* females fed at the surface layers and returned to deeper waters after feeding. Gut fullness decreased rapidly after midnight and returned to low levels at 0800 h. Results of two-way ANOVA showed that the gut fullness of female *P. concinna* differed significantly between day and night (*p* < 0.001), but did not vary among the depth layers (*p* > 0.05).

**Figure 8:**
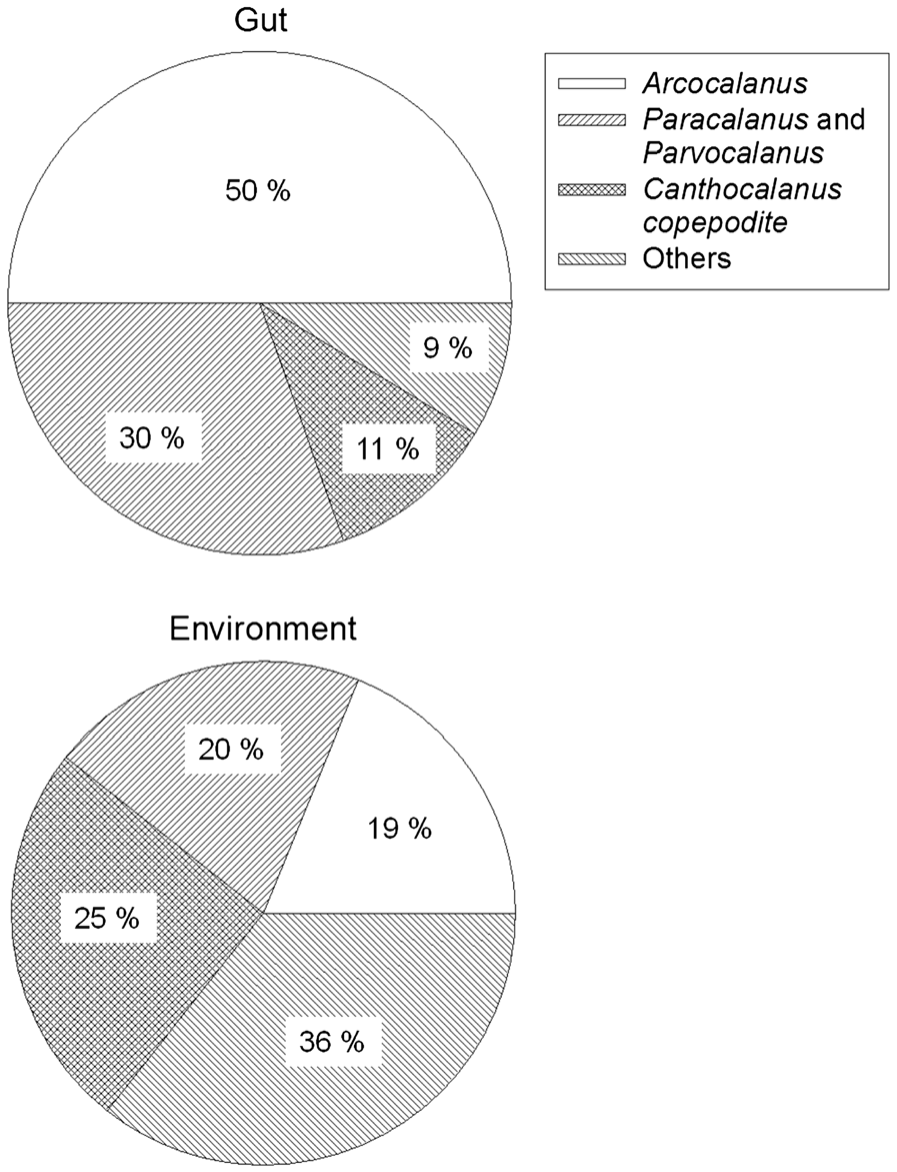
**Composition of copepods in the gut of female *P. concinna *and in the environment.**

Copepods, the major prey of female *P. concinna*, comprised ~ 40% of the gut contents (Figure 8). Eggs, remains of crustaceans and unidentified items made up the remaining gut contents. All copepods found in the gut were calanoid copepods. *Acrocalanus* constituted ~ 50% of all copepod prey. Other common copepod prey included *Paracalanus/Parvocalanus* (~ 30%) and copepodites of *Canthocalanus* (~ 11%). The electivity index (ε) showed that *Acrocalanus* (ε = 0.51) and *Paracalanus/Parvocalanus* (ε = 0.21) were the only prey items that were positively selected by female *P. concinna.*

The mean digestion times of female *P. concinna* were 5.3 h for *Paracalanus/Parvocalanus* and 5.6 h for *Acrocalanus*. The estimated feeding rates of female *P. concinna* ranged from 0 to 0.30 prey predator^-1^h^-1^ on *Acrocalanus* and 0 to 0.38 prey predator^-1^h^-1^ on *Paracalanus/Parvocalanus*. Predation impact, expressed as the percentage of the standing stock removed daily by *P. concinna*, was 4.30% for *Acrocalanus* and 4.26% for *Paracalanus/Parvocalanus*.

### Prey availability

*Acrocalanus* and *Paracalanus/Parvocalanus*, the most ingested and only positively selected prey of *P. concinna* females, stayed nearer to the surface than *P. concinna* females during both day and night, as shown by their WMDs (Table 1). *Acrocalanus* and *Paracalanus/Parvocalanus* did not exhibit DVM, as both WMD and percentage of the population at the surface did not differ significantly between day and night (*p* > 0.05). The WMDs of *Acrocalanus* and *Paracalanus/Parvocalanus* differed significantly (*p* < 0.05) from that of *P. concinna* females only during the day. In contrast, the vertical distribution of *Canthocalanus* copepodites, which were not positively selected by *P. concinna*, overlapped with that of *P. concinna*. Their WMD appeared to be very similar to that of *P. concinna* females during both day and night (Table 1). *Canthocalanus* copepodites exhibited weak DVM, as significant diel difference was found only in the percentage of the population at the surface (*p* < 0.05), but not in WMD (*p* > 0.05).

## Discussion

### *P. concinna* seasonal and spatial distribution

*P. concinna* is the most abundant Euchaetidae in the study area, comprising more than 70% of the total Euchaetidae population (Wong unpublished data). It is one of the four species of Euchaetidae (*P. concinna*, *E. rimana*, *P. plana* and *E. indica*) recorded in Hong Kong’s coastal waters [20,24,32,33]. Along the Chinese coast, *P. concinna* has been reported from the East China Sea, the Taiwan Strait and the South China Sea [18,19,34]. The species is considered to be limited to the Indo-Pacific in distribution [1].

Our results are in agreement with previous studies that dense populations of *P. concinna* appear only in winter and spring in Hong Kong [20,22,32,33], when the water temperature is low (16–24°C). In comparison, Euchaetidae in the temperate oceans of the East China Sea and the Yellow Sea exhibits an opposite seasonal pattern. Both *P. concinna* and *E. rimana* are carried by the Kuroshio Current into the coastal areas of the East China Sea, and reach peak densities in summer and autumn [18]. *P. concinna* occurs throughout the year in the northern Taiwan coast which is influenced by the Kuroshio Current year-round [35], but their density is higher in autumn and winter when water masses from the East China Sea flow toward Taiwan [35].

The abundance and distribution of copepods are known to be influenced by hydrographic conditions [36,37]. The density of *P. concinna* is lower in the semi-enclosed Tolo Harbour than the east and southeastern waters that are fully exposed to water currents from the South China Sea. The low densities at most times of the year, together with the pattern of shoreward decrease in density, suggest that *P. concinna* is transported into the coastal seas of eastern Hong Kong by water currents from the South China Sea, and the scarcity of *P. concinna* in the inner parts of Tolo Harbour can be explained by loss during physical transport.

On the other hand, trophic interactions may also influence the abundance of *P. concinna* in Tolo Harbour. The copepod community in Tolo Harbour is dominated by small copepods such as *Parvocalanus crassirostris* and *Paracalanus parvus*[26]. Such a plethora of small calanoid copepods suggests that carnivorous copepods such as *P. concinna* will not be limited by food [8,9,38,39]. Chang *et al.*[40] proposed that the density of large copepods, such as *P. concinna*, is controlled directly by fishes, while the density of small copepods is controlled by invertebrate predators. Several observations suggest that fish predation may contribute to the scarcity of large copepods such as *P. concinna* in Tolo Harbour and other nearshore areas of Mirs Bay. First, dense populations of the planktivorous *Ambassis* (glassfish) occur in Tolo Harbour throughout the year [41]. Secondly, copepods are known to be the major prey of larval and juvenile fishes which appear in large numbers in Tolo Harbour during late winter and early spring [42].

### Diel behaviors

Both male and female *P. concinna* exhibit DVM. The congener *Paraeuchaeta norvegica* has been observed to perform DVM over depths of hundreds of meters [43-45], but there are also reports of non-migrating populations of this species [12,16]. The vertical distribution of *P. norvegica*[14] and *Paraeuchaeta elongata*[10,46] has been shown to be affected by ovigerity. The reproducing females of the calanoid copepod *Eurytemora hirundoides* have also been found to occupy deeper waters in the Archipelago Sea [47]. Ovigerous *Eurytemora* are highly selected by the planktivorous Baltic herring [48], and Vuorinen [49] has proposed that visual predation is the most probable cause of the difference in vertical distribution between ovigerous and non-ovigerous females. However, the results of this study show that both ovigerous and non-ovigerous females of *P. concinna* perform DVM.

DVM in crustacean zooplankton is widely considered to be an antipredator behavior [15,45,50-53]. Small pelagic organisms have no means to hide from visual predators, such as fish, in well-lit surface waters during the daytime, and must minimize predation risk by seeking refuge in deeper depths that have less light penetration [54]. Light is, therefore, commonly considered as a proximal cause or signal for zooplankton DVM [55,56]. Light intensity drops dramatically in the first 5 m, and very little light penetrates into waters below 15 m. The WMD of *P. concinna* is below 15 m during daytime. This suggests that *P. concinna* adjusts its vertical distribution to achieve an optimal balance between the energy cost of DVM and the benefit of reducing visual predation risks. Vuorinen [49] suggested that it may be a viable strategy for copepods to stay in deeper waters at all times if the temperature and food supply do not vary greatly between the deep and surface layers. While there was little temperature change with depth in the well-mixed, shallow waters of eastern Hong Kong, more than 50% of the *Acrocalanus* and *Paracalanus/Parvocalanus* populations remained in the upper 0–5 m during both day and night, so the need for food may force *P. concinna* females to ascend to the surface at night. On the other hand, while adult male *P. concinna* do not feed, they still perform DVM and migrate to the surface at night. Such DVM behavior may therefore be an intrinsic behavior not driven by the need to feed, but other factors such as light. It is also possible that the *P. concinna* males follow the migrating females to increase the chance of mating, as male copepods of various species, e.g. *Temora longicornis, Centropages typicus, Pseudocalanus elongatus*, and *Acartia tonsa*, have been found to track and pursue female copepods through pheromones or hydromechanical signals for mating [57-59].

Diel patterns in gut fullness suggests that female *P. concinna* only feed actively at night. Diel feeding rhythms have been reported in *Paraeuchaeta* in both laboratory [39] and field studies [13,16]. Marked increase in the gut fullness of female *P. concinna* appears to coincide with their nocturnal ascent in the early part of the night, so the prevalence of individuals with relatively empty guts during the day may be due to a lack of access to prey. On the other hand, diel variations in gut fullness may not be associated with DVM. Female *P. concinna* stay in significantly deeper waters than *Acrocalanus* and *Paracalanus/Parvocalanus* during the day, but appear to share the same depths with *Canthocalanus* copepodites during both day and night. The absence of *Canthocalanus* in the guts of female *P. concinna* in daytime therefore suggests that the predators feed only at night, and implies that the diel variations in gut fullness was not the result of the predators migrating into and out of the prey-rich surface layer. Other studies have found that copepods exhibit diel variations in the gut contents regardless of whether they perform DVM. The gut fluorescence of both surface dwelling and migrating zooplankton in the Bedford Basin reaches peak values only at night [60]. Diel feeding rhythm in two species of *Calanus* in the Bering Sea is also independent of DVM [61], and though *Calanus pacificus* in Dabob Bay performs DVM and enters into the surface layer 2.5 h before sunset, its gut pigment content only increases substantially after sunset [62].

Diel feeding rhythm in zooplankton is often regarded as a strategy to avoid the accumulation of pigments in daytime [62,63], the purpose of which is to reduce the risk of attack by visual predators. Laboratory and field experiments with the rainbow trout *Salmo gairdneri* have confirmed that visual predators select the most pigmented calanoid copepods as prey [64]. Bollens and Stearns [65] reported that the gut fullness of *Acartia hudsonica* is lower in the presence than in the absence of fish. *A. hudsonica* and *Acartia tonsa* reduce their gut fullness in the presence of fish exudates, and the response is observed only when the light level is sufficient for visual predation [66]. These results suggest that *P. concinna* females may stop feeding during the day to reduce the chances of being attacked by fishes.

### Prey composition and selectivity

Small calanoid copepods constitute ~40% of the prey in the guts of female *P. concinna*. The estimate is considered to be conservative as some unidentified items found in the guts may also be the remains of calanoid copepods. The importance of calanoid copepods as food for female *P. concinna* is in accordance with previous findings on the natural diets of *Paraeuchaeta* species [9,38]. Copepods including *Metridia gerlachei*, *Calanoides acutus*, *Euchaeta* spp., *Oncaea* spp., and *Oithona* spp. made up 80–90% of the prey consumed by adults and copepodites (C5) of *Paraeuchaeta antarctica* in Antarctic waters [9]. Øresland and Ward [38] also reported that copepods form 46–99% of the diets of *P. antarctica*, *Paraeuchaeta farrani*, *Paraeuchaeta rasa*, and *Paraeuchaeta biloba* in South Georgia.

*P. concinna* females feed on a variety of small calanoid copepods including *Canthocalanus*, *Centropages*, and *Subeucalanus*, but show strong preference for *Acrocalanus*, *Parvocalanus*, and *Paracalanus*. In laboratory feeding experiments, Yen [67] found that prey size is an important factor in the dietary selectivity of *P. norvegica*. The prosome length of preferred copepod prey is usually ~70% the length of the second basipodal segment of the maxilliped of the predator. A similar proportion of 65% was found for *P. antarctica*, which exhibits the highest feeding rates on copepods with prosome length of 1.2 mm [8]. In this study, the prosome length of *Acrocalanus* (~0.6–0.7 mm) is about 65–75% the length of the second basipodal segment of the maxilliped (~0.92 mm) of *P. concinna* females, agreeing with the optimal prey size proposed by Yen [44]. On the other hand, the prosome lengths of *Paracalanus* and *Parvocalanus* (~0.3–0.4 mm) are only 33–40% the length of the second basipodal segment of the maxilliped of *P. concinna*. These smaller copepods may therefore be more suitable prey for the smaller late copepodite stages of *P. concinna* (C4 and C5). The prosome length of the negatively selected *Canthocalanus* copepodites (~0.8–1.0 mm) is ~85% the length of the second basipodal segment of the predator maxilliped.

*P. concinna* females in this study did not feed on cyclopoid copepods, even though small cyclopoid copepods including *Oithona* and *Oncaea* were common in the gut of *P. antarctica* and may be the major prey of this predatory copepod, especially the copepodite stages [9,38,68]. Yen [67] reported that the cyclopoid copepods *Oithona* and *Corycaeus* are not preferred prey of *P. antarctica*, and proposed that the intermittent and darting movement of cyclopoid copepods are not easily detected by tactile predators. The copepod community in Mirs Bay is dominated by calanoid copepods (> 80% of the total copepod population), and *Oithona* and *Oncaea* species only comprise ~ 7% and 5% respectively of the total copepod populations [25]. The reason for the absence of cyclopoid copepods in the gut of female *P. concinna* may be a combination of the prey’s low abundance, small size (prosome length ~0.2–0.3 mm), and less detectable swimming behavior.

### Digestion time and feeding rate

Tönnesson *et al*. [6] found a gut evacuation rate of 0.080 h^-1^ or digestion time of 12 h at 15°C for *P. norvegica*. This is much longer than the digestion time of ~ 5 h recorded at 18°C for *P. concinna* in this study. The relationship between the predator size and digestion time in copepods is unclear, although some studies have found no relationship between digestion time and predator size [69,70]. A common conclusion from previous studies is that digestion time varies with temperature [71-75] and prey size [76]. The higher water temperature in the subtropical seas of Hong Kong may allow shorter digestion times for female *P. concinna*, but it can also be argued that *Paracalanus* and *Pseudocalanus*, the copepod prey used to estimate the digestion time of *P. norvegica*[6], are bigger than *Acrocalanus* and *Paracalanus/Parvocalanus*, the natural prey of *P. concinna*. On the contrary, Yen [77] reported a considerably higher gut evacuation rate of 0.43 h^-1^ and correspondingly very short digestion time of ~ 2 h for *P. norvegica* feeding on cod larvae at 7.5°C. As fish larvae lack the chitinous exoskeleton of copepod prey, they may be more easily digestible.

While the daily feeding rate of copepods changes with temperature and prey availability [35,71-73], our estimated daily feeding rates for female *P. concinna* feeding on *Acrocalanus* (~4.7 prey predator^-1^d^-1^) and *Paracalanus/Parvocalanus* (~4.4 prey predator^-1^d^-1^) are within the range of values reported for *P. norvegica*. Olsen *et al*. [78] reported a feeding rate of 3.6 prey predator^-1^d^-1^ at 7–10°C in the laboratory. Tönnesson *et al*. [6] reported *in situ* feeding rates of 1.4–5.2 prey predator^-1^d^-1^ at 5°C and 6.2–8.6 prey predator^-1^d^-1^ at 15°C.

### Predation impact

*P. concinna* females remove ~ 4.3% of both the *Acrocalanus* and *Paracalanus/Parvocalanus* standing stock daily in the coastal sea of eastern Hong Kong. The estimates were conservative as only predation by adult females was considered. Øresland [9] found that the feeding rates of copepodites are higher than that of adults in Antarctic waters. The predation impact by the entire *P. concinna* population may therefore be higher as *Paracalanus* and *Parvocalanus* are small enough to be eaten by late copepodite stages, which are frequently more abundant than adults (Wong unpublished data). Predation impact estimated for female *P. concinna* in this study is within the range of values reported for other predatory copepods. Tönnesson *et al*. [6] reported a predation impact of 2.0–6.5% on small copepods by *P. norvegica* in the Skagerrak. The predation impact of *Tortanus* spp. on small copepod populations ranged from 1% in San Franscisco Estuary [79], to 2.7% in Fukuyama Harbour [80].

Copepods are also the major prey of chaetognaths. While the predation impact of chaetognaths on copepods can reach 6% in Northern Chile [81] and 7.8% in the eastern Mediterranean [82], a recent study conducted in Tolo Harbour and Mirs Bay showed that the predation impact of chaetognath on the copepod population is < 1% [83]. This finding suggests that *P. concinna* may play a more important role than other invertebrate predators in regulating the populations of small calanoid copepods in the eastern waters of Hong Kong, especially during winter and spring.

## Conclusions

*P. concinna* is common in the coastal waters of eastern Hong Kong only during winter and spring. A trend of shoreward decrease in abundance suggests that the population in the shallow areas of eastern Hong Kong is derived from populations in offshore areas in the South China Sea. The entire adult population of *P. concinna* remains in dark, deeper waters during the day, probably to minimize the risks of fish predation. At night, *P. concinna* migrate to the surface, where their preferred prey, *Acrocalanus, Paracalanus,* and *Parvocalanus* stay throughout the day. *P. concinna* females with full guts are most common at night, but the diel feeding rhythm is probably not related to DVM. *P. concinna* removes ~4.3% of the *Acrocalanus, Paracalanus,* and *Parvocalanus* standing stock daily, making it an important predator of small calanoid copepods.

## Abbreviations

DVM: Diel vertical migration; WMD: Weighted mean depth.

## Competing interests

The authors declare that they have no competing interests.

## Authors’ contributions

CKW was the Principal Investigator responsible for conceiving, coordinating, obtaining the funding to carry out the study, and modified the final draft of the manuscript. EYWY carried out the experimental work, prepared and analyzed the data, and wrote the first draft of the manuscript. AAYL analyzed and interpreted the data and results, and finalized the manuscript. All the authors read and approved the final manuscript.
